# Dielectric-Masked Selective-Area Porous GaN Microarrays with Embedded Quantum Dots for Color Conversion

**DOI:** 10.3390/nano16181134

**Published:** 2026-09-10

**Authors:** Jaeyoung Baik, Je-Sung Lee, Suhyeon Lee, Jeongtae Kim, Jaeyong Kwon, Jeongwoon Kim, Seung Hyeok Lee, Hoe-Min Kwak, Chang-Mo Kang, Dong-Seon Lee

**Affiliations:** 1 Department of Electrical Engineering and Computer Science, Gwangju Institute of Science and Technology (GIST), 123 Cheomdangwagi-ro, Buk-gu, Jeonnam-Gwangju 61005, Republic of Korea; baikjy409@gist.ac.kr (J.B.); jslee136@gist.ac.kr (J.-S.L.); tngus3918@gm.gist.ac.kr (S.L.); jjeongtae@gm.gist.ac.kr (J.K.); dragon0926@gm.gist.ac.kr (J.K.); jwkim3523@gm.gist.ac.kr (J.K.); lsh06131@gm.gist.ac.kr (S.H.L.); 2 R&D Strategy & Innovation Division, Gwangju Institute of Science and Technology (GIST), 123 Cheomdangwagi-ro, Buk-gu, Jeonnam-Gwangju 61005, Republic of Korea; khm90@gist.ac.kr; 3 Department of Nanomechatronics Engineering, Pusan National University, Busandaehak-ro 63beon-gil, Geumjeong-gu, Busan 46241, Republic of Korea; 4 Department of Semiconductor Engineering, Gwangju Institute of Science and Technology (GIST), 123 Cheomdangwagi-ro, Buk-gu, Jeonnam-Gwangju 61005, Republic of Korea

**Keywords:** quantum dot, porous GaN, color conversion

## Abstract

In this study, we present the development of selective-area porous GaN (SPG) microarrays integrated with embedded quantum dots (QDs) for next-generation display applications. We report a dielectric hard mask-based architecture that withstands high etching voltages without suffering structural damage, overcoming the limitations of conventional polymer masks. This process provides precise control over both vertical and lateral pore propagation, achieving an effective pixel size of 15 μm and realizing an ultrahigh-resolution (>1270 ppi) display structure. Furthermore, optical performance measurements confirmed that the obtained array exhibited an excellent color gamut, reaching 119.2% of the NTSC and 95.6% of the Rec. 2020 standards. Therefore, the present dielectric hard mask-based patterning technology provides an effective solution for achieving both process stability and high-resolution pixel structures, enabling the fabrication of high-performance micro-LED displays with full-color capabilities.

## 1. Introduction

Micro-LEDs (μ-LEDs) have attracted significant attention as next-generation light sources for high-resolution near-eye displays, such as augmented reality (AR), virtual reality (VR), and extended reality (XR) systems, owing to their fast response, long lifetime, and superior luminance [1,2,3]. Near-eye displays require a high pixel density to prevent the screen door effect induced by their short viewing distance, making the heterogeneous integration of μ-LEDs with Si-based driving circuits an essential requirement [4,5,6]. For this purpose, LED-on-silicon (LEDoS) technology has been widely adopted as a representative integration platform [6]. However, current LEDoS technologies rely on high-quality monochromatic LED wafers, making full-color operation challenging with a single panel [3,7]. Hence, full-color displays require three separate RGB LEDoS panels, along with a complex optical system for color combination, which results in high manufacturing costs and a bulky system architecture [8]. To address these limitations, color conversion via colloidal quantum dots (QDs) directly embedded on a single UV or blue μ-LED array has emerged as a viable solution, providing a streamlined process and reduced manufacturing costs [9,10,11].

However, the decreased QD sub-pixel size in ultrahigh-resolution displays creates some serious technical challenges in the application of these color conversion structures. First, increasing the thickness of the color conversion layer to suppress blue light leakage causes additional reabsorption losses between QDs due to their excessive usage, which in turn leads to degradation of the color conversion efficiency [12]. Second, as the device size decreases, achieving uniform quantum dot patterns within the target area becomes highly challenging. Specifically, the coffee ring effect in standard solution-based methods leads to QD aggregation near the boundaries during solvent evaporation, resulting in poor emission uniformity across the sub-pixels [13,14,15].

To overcome these limitations, color conversion structures based on porous GaN have attracted significant attention [16,17,18,19,20]. Multiple light scattering inside the pores increases the optical path length, significantly improving the color conversion efficiency with a smaller amount of QDs in a thinner layer [21]. Furthermore, the petal effect, which arises from the combination of surface hydrophobicity and strong liquid droplet adhesive forces, facilitates uniform QD loading within the pores, thereby yielding highly uniform emission properties [22]. Initial studies on color conversion based on porous GaN were limited to a pixel resolution of 35 μm [23,24,25]. Huang et al. achieved a significant downscaling to the micrometer level by employing a polymer mask [26]. However, this method for pixel size reduction using a polymer mask presents critical limitations. Structural breakdown and delamination of the polymer mask may occur at the high voltages required by the electrochemical etching (ECE) process. Consequently, the etching voltage must be reduced to a low value of 15 V or less. This decreased voltage limits the pore size and porosity of the resulting GaN, leading to a lower color conversion efficiency. Improving the latter requires a thicker porous GaN layer, which increases the overall thickness of the color conversion structure.

To overcome the process limitations stemming from the instability of polymer masks, in this work we present a selective-area porous GaN (SPG) architecture based on a dielectric hard mask, exhibiting high durability under high-voltage ECE conditions. Unlike previous approaches that were limited to low etching voltages or large-scale patterns, our dielectric hard mask technique can withstand a high etching voltage of 22.5 V without structural breakdown. This approach achieves a high porosity, providing enhanced multiple light scattering and color conversion efficiency. Furthermore, upon establishing a minimum 5 μm mask aperture and subsequent selective full-color (RGB) QD infiltration, we successfully obtained a monolithic full-color microarray with an effective pixel size of 15 μm and an ultrahigh resolution of 1270 ppi. This strategy not only addresses existing process limitations, but also paves the way for high-density pixel integration.

## 2. Experimental Methods

### 2.1. Fabrication of SPG and Quantum Dot Integration

**Figure 1:**
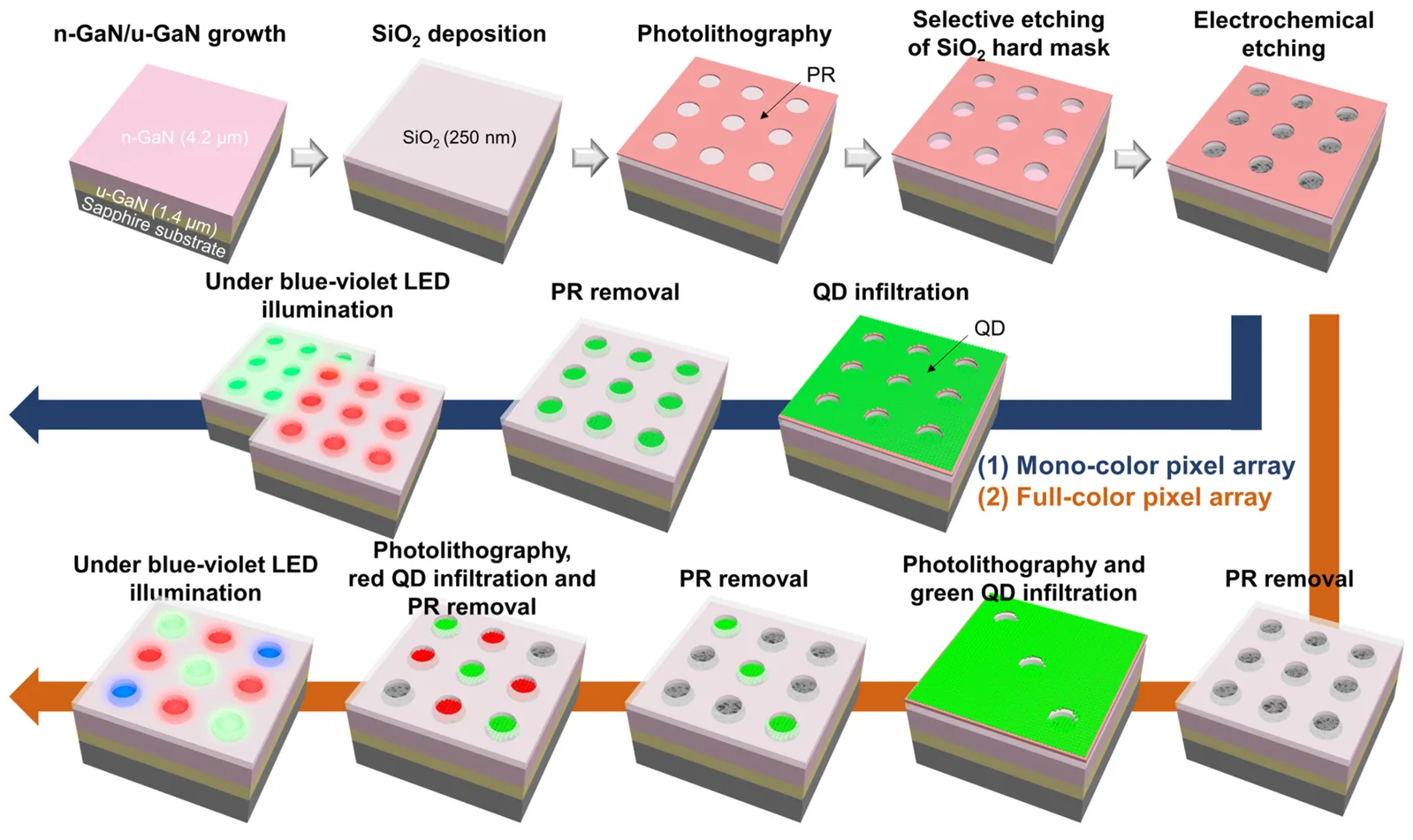
Schematic illustration of fabrication process of QD-integrated SPG pixel arrays. The blue and orange arrows represent the fabrication routes for mono-color and full-color pixel arrays, respectively.

Figure 1 illustrates the fabrication process of the QD-embedded SPG microarray. First, using metal–organic chemical vapor deposition (MOCVD, Marble 260s, Sysnex Co., Ltd., Daejeon, Republic of Korea), a 1.4 μm-thick un-doped GaN (u-GaN) layer and a 4.2 μm-thick n-GaN layer were sequentially grown on a c-plane sapphire substrate polished on both sides to form the n-GaN/u-GaN template, with the n-GaN layer having a silicon doping concentration of 6 × 10^18^ cm^−3^. Next, a 250-nm SiO_2_ film was deposited using plasma-enhanced chemical vapor deposition (PECVD, PlasmaPro 800Plus, Oxford Instruments, Bristol, UK). Afterward, photolithography was used to form a photoresist (PR) pattern with a microhole array structure. To etch the exposed SiO_2_, selective dry etching via inductively coupled plasma reactive ion etching (ICP-RIE, TE3100, TAINICS, Suwon, Republic of Korea) was carried out using CF_4_ gas. To fabricate the SPG structure, ECE was carried out on the exposed n-GaN region. The ECE process was conducted in a 0.3 M oxalic acid (98%, Sigma-Aldrich, St. Louis, MO, USA) aqueous solution under a constant applied voltage of 22.5 V.

To fabricate mono-color pixel arrays via the pathway marked by a blue arrow in the figure, a QD/toluene dispersion with a concentration of 5 mg/mL was drop-cast onto the surface to infiltrate the QDs into the pores, followed by complete drying at room temperature. To ensure a sufficient density of the embedded QDs, the drop-casting and drying process was repeated three times. Finally, a lift-off process using an acetone solution was performed to remove the PR along with any residual QDs, yielding a SPG pixel microarray with the QDs uniformly filling the pores.

To prepare full-color arrays (through the orange arrow-marked pathway), we developed a selective infiltration process involving multiple steps. First, the initial photoresist was removed after ECE to expose the SiO_2_ mask. Then, a second photolithography step opened the target green sub-pixels for green QD drop-casting, followed by a lift-off process. This same sequence was then repeated in a third photolithography step to selectively embed red QDs into the designated red sub-pixels. Finally, a monolithic R/G/B microarray was configured by allowing the remaining uninfiltrated porous GaN area to transmit blue excitation light as intrinsic blue sub-pixels.

### 2.2. Structural and Optical Characterization

The surface and cross-sectional morphologies of the unpatterned and SPG samples after ECE were observed using scanning electron microscopy (SEM; SU5000, Hitachi High-Tech Corp., Tokyo, Japan). The absorption spectra of the unpatterned and porous GaN samples were measured using a UV–Vis–near-infrared spectrophotometer (Cary 5000, Agilent Technologies, Santa Clara, CA, USA). Cross-sectional energy-dispersive X-ray spectroscopy (EDS) elemental mapping was performed using SEM (SU8100, Hitachi High-Tech Corp., Tokyo, Japan). Photoluminescence (PL) measurements were carried out at room temperature using a spectrometer (FLAME-S, Ocean Optics, Orlando, FL, USA), with the QD-embedded porous GaN samples excited by a 410 nm LED light source. Optical microscopy images were acquired using an digital microscope (DSX1000, Olympus Corp., Tokyo, Japan). The localized PL intensity was extracted from the optical images and analyzed using the ImageJ software (ver. 1.54g).

## 3. Results and Discussion

### 3.1. Structural and Optical Characterization of Unpatterned Porous GaN

Prior to characterizing the SPG, we investigated the ECE process on unpatterned structures to elucidate the pore formation mechanism as a function of the applied voltage, which determines the color conversion properties. For this purpose, the ECE process was directly performed on the unpatterned n-GaN templates. The electrochemical n-GaN etching reaction in an oxalic acid electrolyte can be described as follows [27,28]:

(1) $$GaN+3H_{2}O+3h^{+}\to Ga{\left(OH\right)}_{3}+\frac{1}{2}N_{2}+3H^{+}\;Ga{\left(OH\right)}_{3}+3H^{+}\to {Ga}^{3+}+3H_{2}O$$

**Figure 2:**
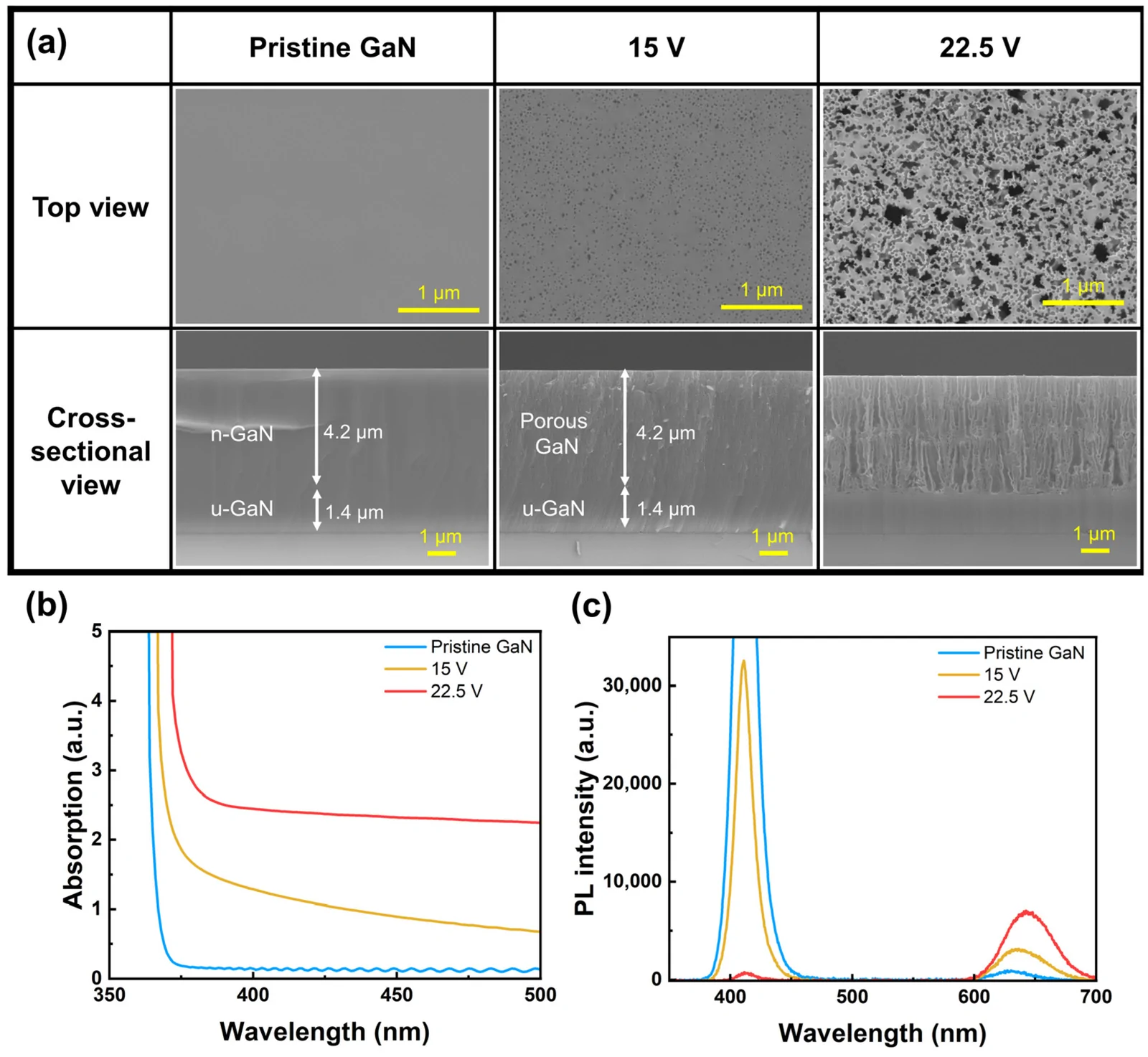
Structural and optical characteristics of unpatterned porous GaN templates: (**a**) surface and cross-sectional SEM images, (**b**) optical absorption spectra of pristine and porous GaN at different voltages, and (**c**) PL spectra of pristine and QD-embedded porous GaN samples.

Figure 2a displays the top-view and cross-sectional SEM images of pristine, 15 V-, and 22.5 V-etched GaN samples. As shown in the top-view image, pristine GaN exhibited a flat surface without any visible defects. In contrast, while the sample etched at 15 V displayed a sparse pore distribution, a dense porous structure was observed at an increased voltage of 22.5 V. Specifically, the porosity increased from 15% to 55% as the applied voltage was raised from 15 to 22.5 V. Simultaneously, the average pore size increased from 30 ± 8 to 72 ± 18 nm, reflecting a broader size distribution (Figure S1). This voltage-dependent structural evolution can be explained by the electrochemical etching kinetics described in Equation (1). At an elevated voltage of 22.5 V, the stronger electric field promotes hole injection at the GaN/electrolyte interface, facilitating the continuous anodic oxidation of GaN and the subsequent dissolution of Ga(OH)_3_ into Ga^3+^ ions. Consequently, the dissolution extends laterally along the pore walls, leading to wider pores and higher porosity. The optical absorption spectra in Figure 2b demonstrate the enhanced light-trapping ability induced by the light-scattering effect within the porous GaN structure. The 22.5 V-etched sample showed the highest absorbance, indicating that the high-porosity structure extends the optical path length via strong multiple scattering. As a result, the internal light is effectively trapped, enhancing the optical interaction with the QDs. Figure 2c shows the PL spectra of the QD-embedded porous GaN samples measured under excitation by a 410 nm LED. In the case of pristine GaN, we observed significant leakage of the external excitation source. Although this leakage remained pronounced for the 15 V-etched sample, its QD emission intensity was higher than that of pristine GaN. Furthermore, etching at a higher voltage of 22.5 V drastically reduced the excitation light leakage and enhanced the QD emission intensity. This optical improvement was accompanied by a redshift in the peak wavelength. As the pore size and porosity increase with the etching voltage, the intensified multiple light scattering within the enlarged porous structure not only enhances the excitation light absorption by the QDs, but also promotes the reabsorption of higher-energy emission photons, which is directly responsible for the observed spectral redshift [22,29]. These findings confirm that the high-porosity GaN structure formed at 22.5 V provides an efficient light-trapping medium based on its light-scattering properties, thereby significantly enhancing the color conversion characteristics.

### 3.2. Structural and Optical Characteristics of SPG Prepared via Dielectric Mask

Based on the above analysis of the planar structures, the high voltage (22.5 V) was confirmed as the optimal processing condition to suppress excitation light leakage and enhance QD emission. Using these established parameters, we fabricated SPG and investigated its structural evolution as a function of the ECE duration at 22.5 V.

**Figure 3:**
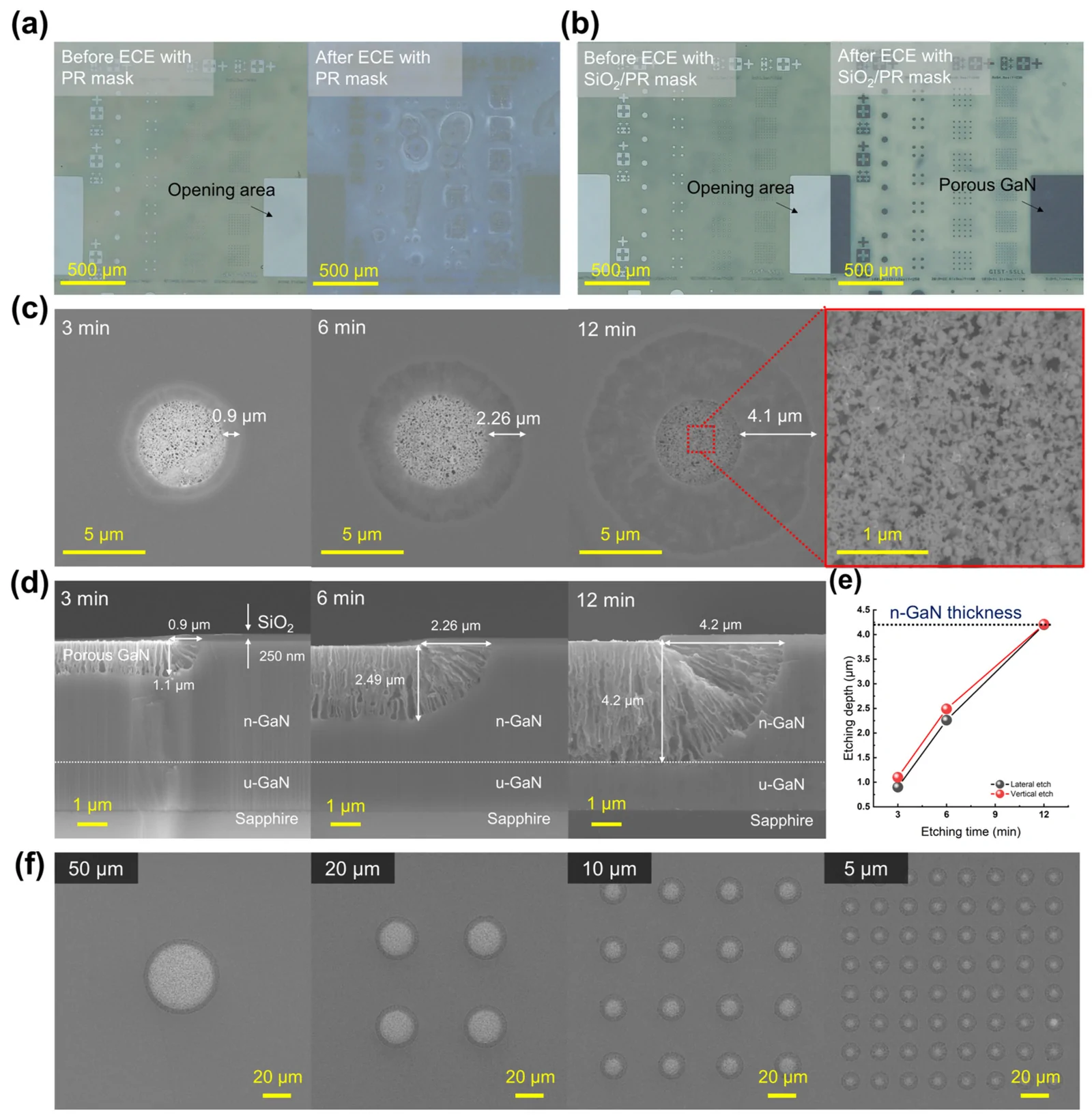
Optical microscopy images showing (**a**) PR-masked and (**b**) SiO_2_/PR-masked n-GaN surfaces before and after ECE. (**c**) Top-view and (**d**) cross-sectional SEM images showing temporal evolution at ECE intervals of 3, 6, and 12 min. (**e**) Plot of lateral and vertical etching depths vs. ECE time. (**f**) Top-view SEM micrographs of fabricated SPG pixel arrays with pixel diameters ranging from 5 to 50 μm. The cross symbols visible in (**a**,**b**) are lithographic alignment marks, and the periodic dots correspond to the patterned microhole arrays.

As mentioned in the introduction, the structural stability and chemical resistance of the mask during the ECE process are critical factors determining the final device quality. Figure 3a compares the optical microscopy images of a PR mask before and after the ECE process. Under the high applied voltage of 22.5 V, the PR mask showed an unstable breakdown of the etching boundaries due to poor interfacial adhesion (Figure S2 shows a photograph of the actual sample). In contrast, in the case of the SiO_2_/PR mask structure, the ECE process took place with high stability without any peeling or mask degradation, even though the PR remained on the top of the structure (Figure 3b).

Next, SEM was employed for further investigating the internal morphology after the removal of the PR mask. The top-view SEM image in Figure 3c confirms the formation of the porous structure inside the microsized hole opening, along with lateral etching simultaneously starting underneath the SiO_2_ mask. High-magnification imaging of the porous region inside the hole confirmed that the corresponding pore size and porosity were consistent with those observed in the conventional planar structure. This indicates that pore formation under the same voltage remained uniform with the planar structure, when ECE is confined within the hard-mask opening.

To quantitatively evaluate the directional etching characteristics, we examined cross-sectional SEM images as a function of the ECE duration (Figure 3d). The SPG sample exhibited an isotropic-like etching behavior with similar lateral and vertical etching rates, as shown by the plot in Figure 3e. The electrochemical reaction initiated at the exposed mask openings induces lateral hole transport within the GaN layer, resulting in anodic oxidation beneath the SiO_2_ mask. Specifically, the dielectric SiO_2_ pattern alters the electric field distribution and the carrier depletion region near the pattern edge, guiding the electric field and hole transport laterally toward the pore walls [30]. The subsequent dissolution of oxidized Ga species by the electrolyte enables the formation of porous structures extending laterally underneath the hard mask. The vertical etching depth reached approximately 4.2 μm after 12 min of ECE, consistent with the thickness of the n-GaN layer. Although the mask pattern size was limited to 5 μm in this study, the final pixel dimension can be precisely controlled by tailoring the mask opening size, taking into account the lateral diffusion and etching characteristics during the ECE process.

Furthermore, porous pixel microarrays featuring opening sizes of 50, 20, 10, and 5 μm were prepared to evaluate the scalability of the present SA ECE process under an etching time of 12 min (Figure 3f). The analysis confirmed that the lateral etching rate remained almost identical across all pixel sizes, ensuring a high process stability. In addition, we systematically evaluated the spatial uniformity across different regions of the sample, showing highly consistent lateral etching lengths regardless of the aperture position (Figure S3). This independence of the lateral etching behavior from pixel size and location indicates that the present technique can be applied to smaller (sub-5 μm) pixel architectures, provided that an insulating SiO_2_ hard mask, uniform n-GaN doping, and efficient current spreading are maintained across the substrate.

**Figure 4:**
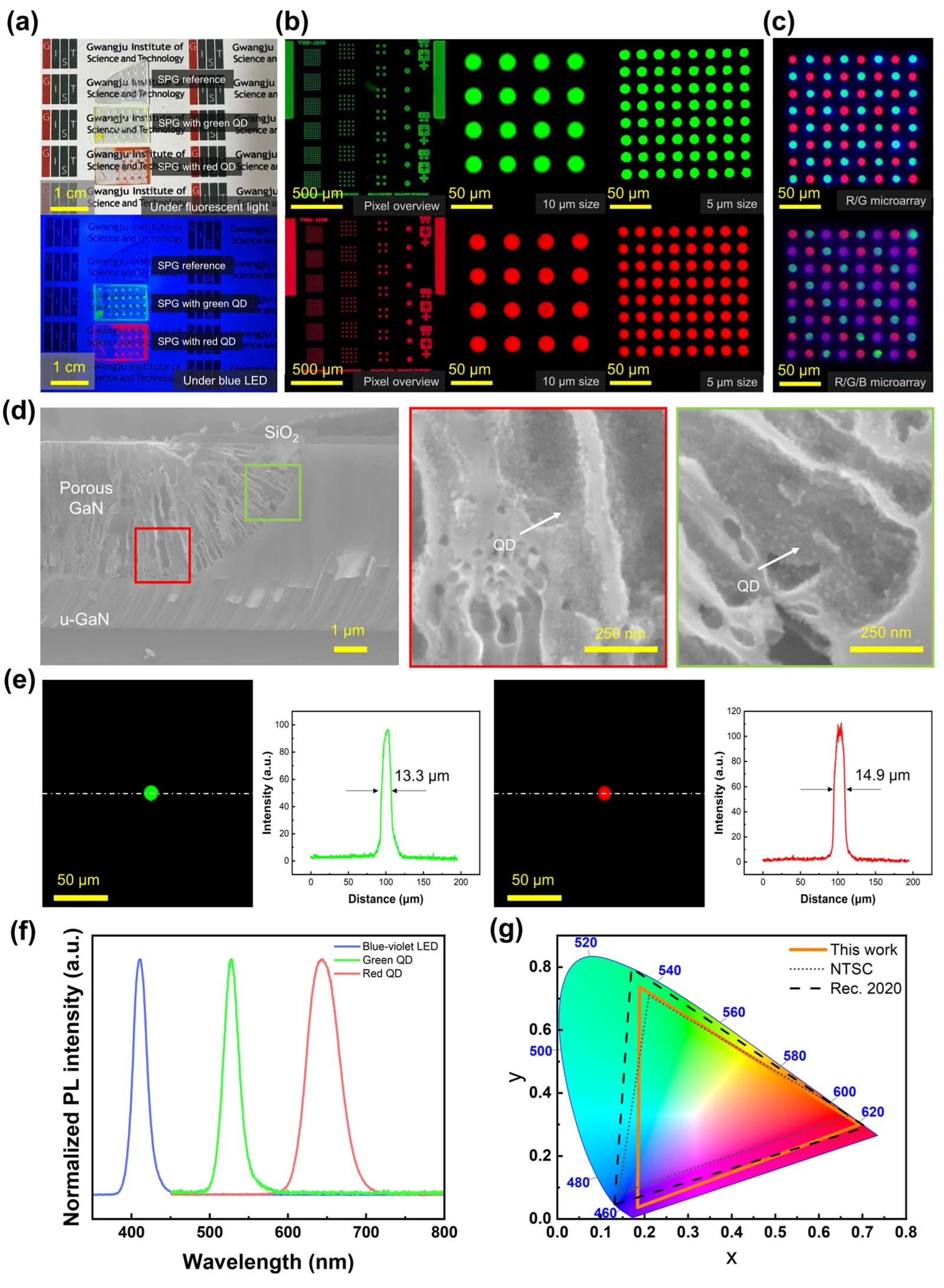
Photoluminescence and structural performance of green and red QD-integrated SPG pixel arrays: (**a**) photographs under ambient fluorescent light and 410 nm blue-violet LED excitation source; (**b**) PL microscopy images of pixel arrays with different geometries; (**c**) microscopy images of 5 μm-patterned red/green (R/G) and red/green/blue (R/G/B) microarrays; (**d**) cross-sectional SEM images confirming QD infiltration in both vertical and lateral pores; (**e**) localized PL intensity profiles and corresponding optical images of a single 5 μm-patterned pixel; (**f**) normalized PL spectra of blue LED and color-converted QDs; (**g**) calculated CIE 1931 chromaticity coordinates compared with NTSC (1953) and Rec. 2020 standards.

Figure 4 presents the results of the optical and structural characterizations performed after embedding green and red QDs inside the pores of the SPG sample. The photographs shown in Figure 4a reveal that the distinctive color emission from each QD is localized within the specific regions and easily distinguishable under both fluorescent light and 410 nm blue-violet LED excitation. Furthermore, large-scale characterizations of the overall pixel array alongside individual pixel arrays from the 5 and 10 μm mask patterns confirmed the successful fabrication of green and red pixel arrays, achieving an ultrahigh-definition of 1270 ppi (Figure 4b). To further demonstrate the feasibility of full-color display integration, PL measurements were conducted on 5 μm-patterned red/green (R/G) and red/green/blue (R/G/B) microarrays (Figure 4c). In the R/G/B microarray, a complete full-color pixel scheme was successfully configured by selectively infiltrating red and green QDs into porous pixels while utilizing the underlying blue excitation light as blue sub-pixels. Each sub-pixel in the microarray exhibited distinct and highly localized emission with clearly defined boundaries, achieving excellent pixel-to-pixel uniformity with a relative standard deviation (RSD) below 5% (Figure S4 and Table S1). Although light propagation from neighboring emitters resulted in an inter-pixel optical crosstalk ratio of approximately 29.5% between adjacent sub-pixels, the strong intensity contrast clearly delineates each individual pixel boundary.

The nanoscale incorporation of the QDs within the pore channels was confirmed via cross-sectional SEM characterization (Figure 4d). The high-magnification image of the vertical porous GaN shows the presence of QDs within the pore structures; similarly, the lateral etching domain observed beneath the hard mask confirmed the successful loading of QDs inside every internal void, which was further supported by cross-sectional EDS elemental mapping (Figure S5). These results demonstrate that the QDs were embedded deep inside the voids during the drop-casting process regardless of the pore orientation, indicating that the extended regions produced by lateral etching could also contribute to the final optical emission.

Therefore, we analyzed the optical emission of a single pixel prepared using the 5 μm mask (Figure 4e). Based on the spatial emission intensity profile along the center of the pixel, the FWHM values for the green and red pixels were determined to be 13.3 and 14.9 μm, respectively; these results confirm that both pixel dimensions were precisely controlled within approximately 15 μm. This indicates that the lateral etching behavior shown by the cross-sectional SEM images was reflected in the spatial distribution of the actual QD emission area. Figure 4f presents the normalized PL spectra of the blue excitation light as well as color-converted green and red QDs. Upon integration of a color filter to remove residual excitation light, the emission data exhibited a superior color gamut, reaching 119.2% and 95.6% of the NTSC (1953) and Rec. 2020 standards, respectively, in the CIE 1931 space (Figure 4g).

To evaluate the technological significance of this work, the present system is compared with previously reported porous GaN-based structures in Table 1. Among investigations employing the cost-effective drop-casting method for QD infiltration, our study achieved the highest ECE voltage of 22.5 V while simultaneously exhibiting the smallest pixel dimension. Importantly, the n-GaN doping concentration of 6 × 10^18^ cm^−3^ used in this work is close to the values (typically around 5 × 10^18^ cm^−3^) reported in the previous studies.

Although the isotropic ECE behavior of the present structure causes lateral etching beneath the mask, increasing the effective emission pixel size to approximately 15 μm, the pixel size can be further reduced by precisely tailoring the mask hole size while taking into account the lateral etching effect. Alternatively, this can be achieved by introducing a light-blocking thin film such as Cr on the SiO_2_ layer to serve as a black matrix structure (see Figure S6 for a schematic illustration of the proposed architecture). Within this architecture, the SiO_2_ hard mask can withstand high-voltage ECE conditions by behaving as a robust protective layer, while any side emission leakage from the laterally etched porous GaN is shielded by the remaining blocking layer, precisely restricting the extracted pixel size to the targeted 5 μm scale. Moreover, for practical applications in high-resolution displays, it is essential to address general engineering challenges such as precise mask overlay, QD photostability, and heat dissipation. Precise mask alignment for sub-5 µm pixels can be achieved using advanced lithography, while the operational photostability and thermal management of the embedded QDs can be ensured via conformal ALD passivation (e.g., Al_2_O_3_) [31,32]. Ultimately, the high-precision QD integration platform presented in this study holds great promise as a key technology for realizing color-converting μ-LED displays with full-color capabilities for next-generation AR/VR and XR applications.

## 4. Conclusions

In this study, we successfully developed a dielectric hard mask-based SPG structure with excellent durability under high-voltage ECE environments, overcoming the low-voltage limitation imposed by the degradation of conventional polymer masks. At a high etching voltage of 22.5 V, a porous structure identical to the planar layer was achieved without mask collapse or delamination. Simultaneously, we obtained an effective lateral porous GaN depth of 4.2 μm, consistent with the thickness of the n-GaN layer. Green and red QDs were successfully infiltrated into the fabricated localized pores via a drop-casting process, confirming that the QDs were embedded within both vertical and lateral pores to realize a pixel-shaped emission. As a result, the 5 μm mask pattern yielded a sub-pixel array exhibiting a spatial FWHM of the luminescence profile of ~15 μm, achieving an ultrahigh resolution of 1270 ppi. Furthermore, upon integration of a simulated color filter, the embedded QD pixel array displayed an outstanding color gamut, reaching 119.2% and 95.6% of the NTSC (1953) and Rec. 2020 standards, respectively. Based on these advantages, the high-voltage SPG/high-precision QD integration technology developed in this work holds great promise for realizing color-converting micro-LED displays with full-color capabilities for future AR/VR and XR applications.

## Figures and Tables

**Table 1:** Comparison of porous GaN-based color conversion structures.

Research Group	Pixel Size	Applied Voltage	Porous GaN Thickness (μm)	Light Conversion Efficiency (%)	Pixel Color	Pixel Isolation Method	QD Infiltration Method	QD Materials Type	Pixels Per Inch (ppi)	Year	Reference
Yale University	36 μm	22.5 V	4.8	100	Full color (R/G/B)	Mesa etching	Drop-casting	CdSe/ZnS QD	508	2020	[23]
Yale University	35 μm	22.5 V	4.8	94	Full color (R/G/B)	Mesa etching	Inkjet printing	CdSe/ZnS QD	508	2020	[24]
National Yang Ming Chiao Tung University & National Taiwan University	20 μm	22 V	3	96	Full color (R/G/B)	Mesa etching	Super inkjet printing	CdSe/ZnS QD	847	2021	[25]
National Taiwan University	<5 μm	15 V	7	<95	Mono-color (R or G)	Black matrix photoresist	Drop-casting	Perovskite QD	5080	2025	[26]
GIST (Our work)	15 μm (scalable to 5 μm)	22.5 V	4.2	98	Full color (R/G/B)	Selective SiO_2_/photoresist	Drop-casting	CdSe/ZnS QD	1270	2026	This work

## Data Availability

The data presented in this study are available on request from the corresponding authors.

## Supplementary Materials

The following supporting information can be downloaded at: https://www.mdpi.com/article/10.3390/nano16181134/s1, Figure S1: Comparison of porous GaN layers fabricated under 15 and 22.5 V ECE conditions: (a) top-view SEM images (left column) and corresponding ImageJ-processed binary thresholded images (right column) for porosity analysis; (b) pore size histograms with fitted distributions for the samples etched at 15 and 22.5 V; Figure S2: Macroscopic surface photographs of GaN templates after short electrochemical etching (ECE) treatment for 1 min at 22.5 V using (top) a PR mask and (bottom) a SiO_2_/PR mask. Complete peeling of the PR layer occurs within only 1 min of etching, whereas the dielectric SiO_2_/PR structure maintains an intact pattern without any film detachment; Figure S3: (a) Optical microscopy images of porous GaN apertures of different sizes (5, 10, and 20 μm) in upper and lower regions. (b) Lateral etching length vs. aperture size across upper and lower sides; Figure S4: Spatial confinement and uniformity analysis of full-color micropixel array: (a) optical fluorescence microscopy image of the array; (b) 2D spatial PL intensity map; (c) line intensity profiles along the horizontal (black) and vertical (red) dashed lines in (b); Figure S5: Cross-sectional EDS elemental mapping (Si, Ga, Al, and S) of the QD-infiltrated porous GaN structure; Figure S6: Schematic illustration of proposed optical confinement strategy using a light-blocking layer; Table S1: Quantitative spatial confinement and emission uniformity parameters of micropixel array extracted from orthogonal line intensity profiles.
