# Supplementary material for: Dielectric-Masked Selective-Area Porous GaN Microarrays with Embedded Quantum Dots for Color Conversion
Source: Nanomaterials (Basel). 2026 Sep 10;16(18):1134. doi: 10.3390/nano16181134 (PMC13609864; doi:10.3390/nano16181134)
Supplement: Supplementary file 1 [file nanomaterials-16-01134-s001.zip › nanomaterials-4498462-supplementary.pdf]

nanomaterials

MDPI

Supplementary Materials

Dielectric-Masked Selective-Area Porous GaN Microarrays with Embedded Quantum Dots for Color Conversion

Jaeyoung Baik [1], Je-Sung Lee [1], Suhyeon Lee [1], Jeongtae Kim [1], Jaeyong Kwon [1], Jeongwoon Kim [1], Seung Hyeok Lee [1], Hoe-Min Kwak [2], Chang-Mo Kang [3,*] and Dong-Seon Lee [1,4,*]

1 Department of Electrical Engineering and Computer Science, Gwangju Institute of Science and Technology (GIST), 123 Cheomdangwagi-ro, Buk-gu, Jeonnam-Gwangju 61005, Republic of Korea; baikjy409@gist.ac.kr (J.B.); jslee136@gist.ac.kr (J.-S.L.); tngus3918@gm.gist.ac.kr (S.L.); jjeongtae@gm.gist.ac.kr (J.K.); dragon0926@gm.gist.ac.kr (J.K.); jwkim3523@gm.gist.ac.kr (J.K.); lsh06131@gm.gist.ac.kr (S.H.L.)
2 R&D Strategy & Innovation Division, Gwangju Institute of Science and Technology (GIST), 123 Cheomdangwagi-ro, Buk-gu, Jeonnam-Gwangju 61005, Republic of Korea; khm90@gist.ac.kr
3 Department of Nanomechatronics Engineering, Pusan National University, Busandaehak-ro 63beon-gil, Geumjeong-gu, Busan 46241, Republic of Korea
4 Department of Semiconductor Engineering, Gwangju Institute of Science and Technology (GIST), 123 Cheomdangwagi-ro, Buk-gu, Jeonnam-Gwangju 61005, Republic of Korea
* Correspondence: fd1kcm@pusan.ac.kr (C.-M.K.); dslee66@gist.ac.kr (D.-S.L.)

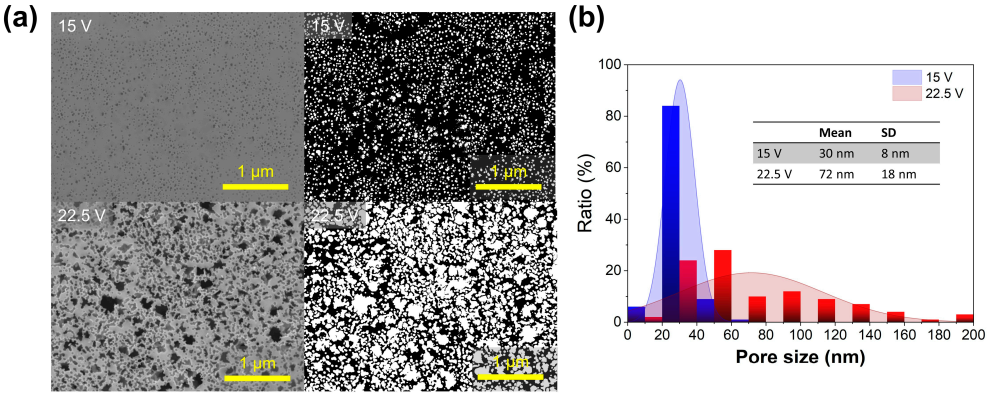

Figure S1. Comparison of porous GaN layers fabricated under 15 and 22.5 V ECE conditions: (a) top-view SEM images (left column) and corresponding ImageJ-processed binary thresholded images (right column) for porosity analysis; (b) pore size histograms with fitted distributions for the samples etched at 15 and 22.5 V.

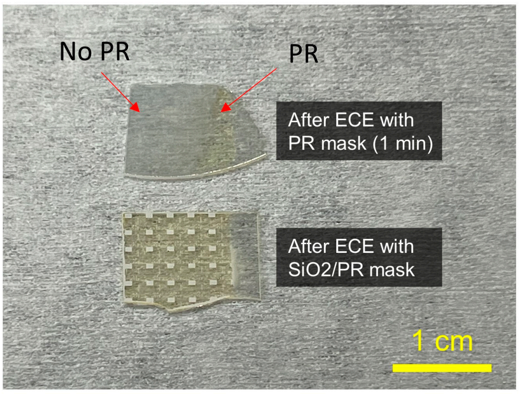

Figure S2. Macroscopic surface photographs of GaN templates after short electrochemical etching (ECE) treatment for 1 min at 22.5 V using (top) a PR mask and (bottom) a SiO_2/PR mask. Complete peeling of the PR layer occurs within only 1 min of etching, whereas the dielectric SiO_2/PR structure maintains an intact pattern without any film detachment.

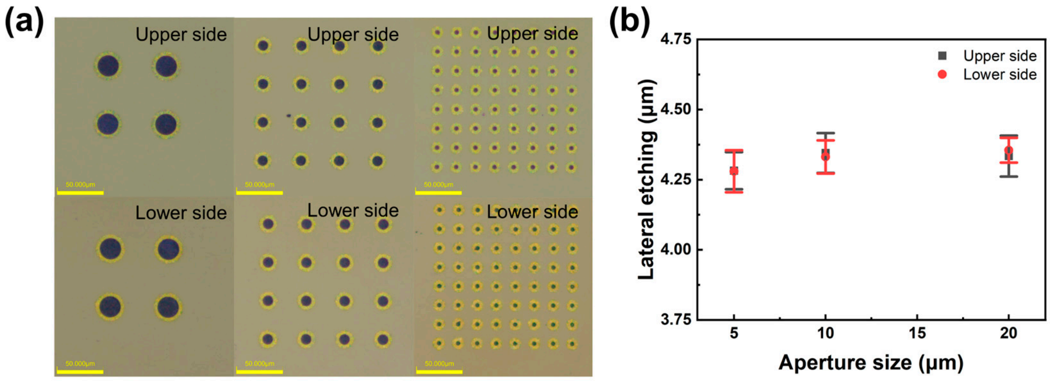

Figure S3. (a) Optical microscopy images of porous GaN apertures of different sizes (5, 10, and 20 μm) in upper and lower regions. (b) Lateral etching length *vs.* aperture size across upper and lower sides**.**

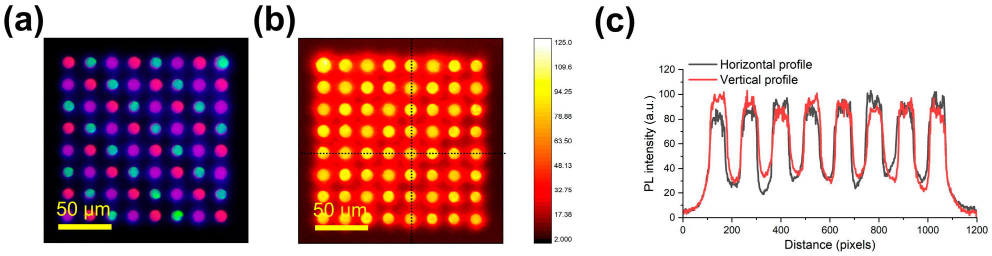

Figure S4. Spatial confinement and uniformity analysis of full-color micropixel array: (a) optical fluorescence microscopy image of the array; (b) 2D spatial PL intensity map; (c) line intensity profiles along the horizontal (black) and vertical (red) dashed lines in (b).

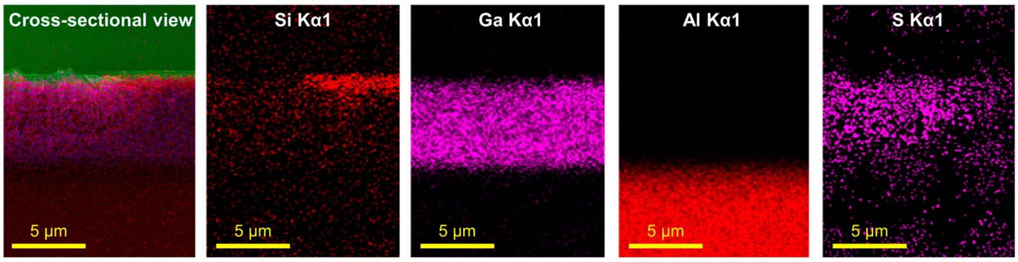

Figure S5. Cross-sectional EDS elemental mapping (Si, Ga, Al, and S) of the QD-infiltrated porous GaN structure.

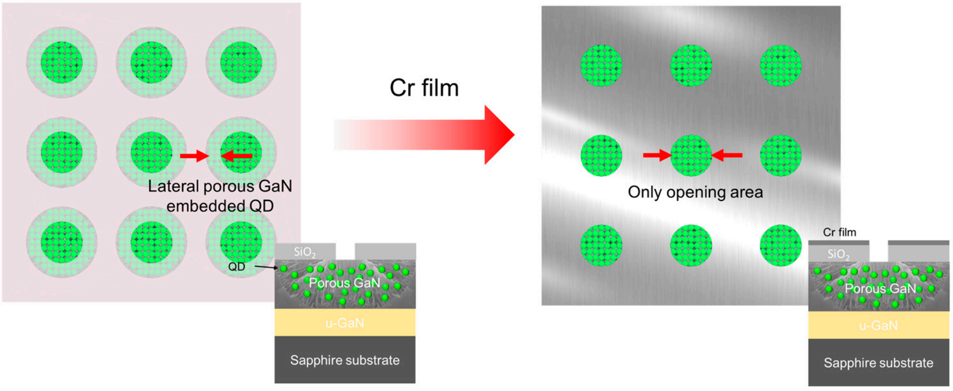

Figure S6. Schematic illustration of proposed optical confinement strategy using a light-blocking layer.

Table S1. Quantitative spatial confinement and emission uniformity parameters of micropixel array extracted from orthogonal line intensity profiles.

Parameter	Horizontal profile	Vertical profile
Mean peak intensity (a.u.)	95.62 ± 5.10	97.25 ± 4.62
Emission non-uniformity (RSD, %)	5.33	4.75
Inter-pixel background intensity (a.u.)	27.43 ± 5.06	29.43 ± 4.58
Inter-pixel optical leakage ratio (%)	28.68	30.26
